# Adipose-androgen crosstalk in polycystic ovary syndrome: mechanisms and therapeutic implications

**DOI:** 10.3389/fendo.2025.1731179

**Published:** 2025-12-16

**Authors:** Muchu Ni, Hongyan Lei, Tao Ye, Yongzhou Wang

**Affiliations:** 1 School of Integrated Traditional Chinese and Western Medicine, Southwest Medical University, Luzhou, Sichuan, China; 2 Department of Gynecology, Gynecology Department of Affiliated Traditional Chinese Medicine Hospital of Southwest Medical University, Luzhou, Sichuan, China

**Keywords:** polycystic ovary syndrome, hyperandrogenism, adipose tissue, insulin resistance, ovulatory dysfunction

## Abstract

Polycystic ovary syndrome (PCOS) is the most common endocrine-metabolic disorder in reproductive-age women, characterized by hyperandrogenism (HA) and insulin resistance (IR). Despite its high prevalence, the underlying pathophysiology remains incompletely understood. In recent years, bidirectional interactions between androgens and adipose tissue (AT) have been recognized as a key driver of the vicious cycle in PCOS. This review systematically examines this core interaction mechanism: on one hand, dysfunctional AT (particularly visceral fat) exacerbates ovarian androgen overproduction by intensifying IR, inducing chronic low-grade inflammation (e.g., elevated TNF-α and IL-6), and reducing adiponectin levels. Conversely, HÀ exacerbates AT dysfunction and systemic IR by altering body fat distribution (central obesity), suppressing lipogenesis, impairing lipolysis, and disrupting adipokine secretion (e.g., reduced adiponectin, elevated leptin). This bidirectional positive feedback loop within the fat-androgen axis perpetuates the worsening metabolic and reproductive abnormalities in PCOS. Based on this mechanism, existing therapeutic strategies—including lifestyle interventions, insulin sensitizers (e.g., metformin), GLP-1 receptor agonists, and anti-androgens—partially exert their effects by improving AT function and antagonizing androgenic effects. Emerging therapies such as SGLT-2 inhibitors, BAT transplantation, anti-TNF-α therapies, and gut microbiota targeting offer promising new avenues for directly intervening in this axis and breaking the vicious cycle of PCOS. A deeper understanding of fat-androgen interactions is crucial for developing precision treatments for PCOS.

## 1 Introduction

Polycystic ovary syndrome (PCOS) is the most common endocrine disorder in premenopausal women. Its prevalence varies depending on diagnostic criteria but generally ranges from 5% to 18% (1) (**Table 1**), being more prevalent in overweight or obese women and specific ethnic groups (2, 3). The clinical hallmarks of PCOS are ovulatory dysfunction, hyperandrogenism (HA), and insulin resistance (IR) (4). Diagnosis currently follows the 2003 Rotterdam criteria, requiring confirmation by two of the following three criteria: HA (clinical or biochemical), irregular menstrual cycles, and polycystic ovarian morphology (PCOM) (5). PCOS increases women’s risk of cardiovascular disease, metabolic syndrome, type 2 diabetes mellitus (T2DM), endometrial cancer, and other conditions (6–8).Beyond physiological manifestations, PCOS is associated with anxiety, depression, eating disorders, sexual dysfunction, and negative body image, leading to reduced health-related quality of life (9). Despite its high prevalence, PCOS remains a complex and understudied disorder. Its etiology is multifactorial, involving interactions between genetic, endocrine, and metabolic factors. Unfortunately, our understanding of the core pathophysiological mechanisms—how these factors synergize to ultimately cause PCOS—remains limited. More notably, the impact of PCOS may extend beyond women, as its pathophysiological basis or associated metabolic dysregulation potentially affects the health of male relatives (10). This high complexity, research lag, and potential widespread implications for both genders’ health collectively contribute to PCOS imposing an increasingly prominent and substantial health and economic burden worldwide. Research indicates that even in non-obese PCOS patients, androgen abnormalities are closely associated with adipose tissue(AT) dysfunction (11), suggesting a tight interaction between the two. Therefore, this review systematically describes how the bidirectional regulation between androgens and AT drives the vicious cycle of PCOS, providing new insights and approaches for understanding its pathophysiology and treatment strategies.

**Table 1:** Diagnostic criteria for polycystic ovary syndrome.

Source (year)	Criteria	Diagnostic threshold
NIH(1990)	①Oligo-anovulation ② Clinical and/or biochemical hyperandrogenism	①②
NIH(1991)	①Oligo-anovulation ② Clinical and/or biochemical hyperandrogenism ③PCOM	Two of three criteria needed
AES(2006)	①Clinical and/or biochemical hyperandrogenism ②Ovarian dysfunction(oligo-anovulation or PCOM)	①②
Japan Diagnostic (2007)	①Menstrual abnormalities( including amenorrhea, oligomenorrhea, or anovulation) ②PCOM ③Clinical and/or biochemical hyperandrogenism	①②③
International evidence-based guideline (2023)	①Oligo-anovulation ②Clinical and/or biochemical hyperandrogenism ③PCOM or increased levels of AMH	Two of three criteria needed

PCOM, polycystic ovarian morphology; AMH, Anti-Müllerian hormone (AMH) is a glycoprotein hormone secreted by small ovarian follicles, primarily produced by granulosa cells in preantral and small antral follicles. It plays a crucial role in reflecting ovarian reserve function, aiding in disease diagnosis, and guiding assisted reproductive technologies. PCOM or AMH levels should not be used in adolescents for diagnosis within 8 years of menarche due to overlap with normal reproductive physiology.

## 2 Classification of AT

### 2.1 Visceral adipose tissue and subcutaneous adipose tissue

**Figure 1:**
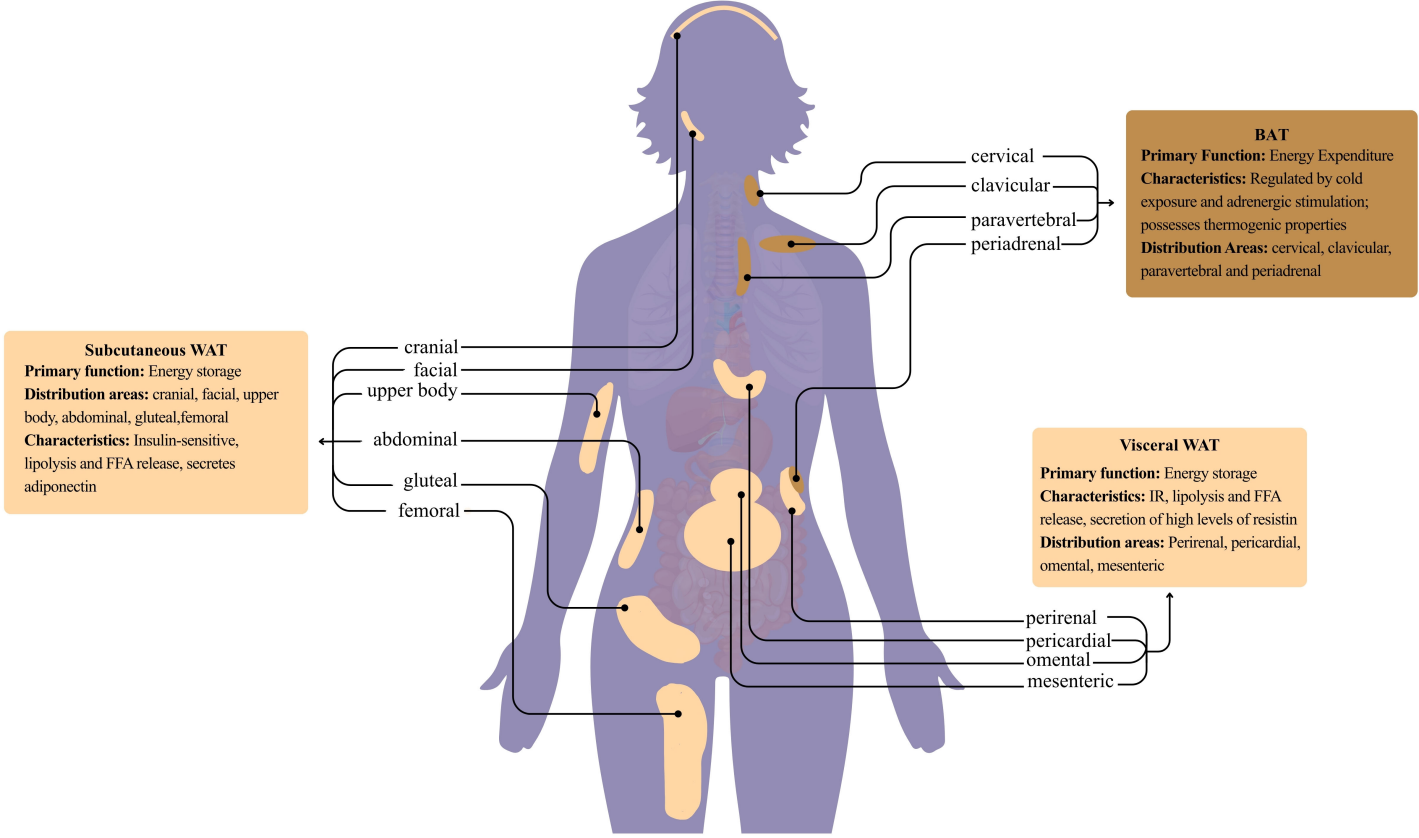
Classification and characteristics of human adepose tissue.

In recent years, growing recognition has emerged that the disease risks associated with obesity and weight gain are more closely linked to the distribution of body fat than to total fat mass (12). As a reservoir for triglycerides, AT exhibits uneven distribution within the body, manifesting as two primary compartments: subcutaneous and visceral (13). Thus, AT can be categorized into SCAT and VAT(**Figure 1**). Small adipocytes in SCAT exert potent buffering effects by uptake of circulating free fatty acids and TGs during postprandial periods. However, if they become dysfunctional in terms of lipogenesis or fat storage capacity within SCAT, fat begins accumulating in tissues ill-suited for lipid storage, such as VAT (14). VAT primarily resides in the mesentery and omentum, directly draining into the liver via the portal venous system. It expresses numerous substances strongly implicated in cardiovascular disease, such as leptin, TNFα, IL-6, and PAI-1, significantly influencing inflammatory processes (15). SCAT deposition occurs primarily in the gluteal region, back, and anterior abdominal wall (16). It exhibits lower metabolic activity than VAT and predominantly produces protective substances like leptin and adiponectin (15). Compared to SCAT, VAT is more cellular, vascular, and innervated, containing more inflammatory and immune cells, less preadipocyte differentiation potential, and a higher proportion of large adipocytes (17). VAT contains more glucocorticoid and androgen receptors than SCAT (18).Compared to SCAT adipocytes, VAT adipocytes exhibit heightened metabolic activity, greater sensitivity to lipolysis, and stronger IR (19). VAT possesses greater free fatty acid production capacity and glucose uptake ability than SCAT, while SCAT demonstrates greater affinity for circulating free fatty acids and triglyceride uptake (20). A study by Dumesic et al. revealed increased VAT in non-obese PCOS patients, correlating with their androgen levels and IR severity (21). Numerous studies confirm VAT as a key mediator of PCOS metabolic and reproductive dysfunction, making its assessment and management central to comprehensive PCOS treatment.

### 2.2 Brown adipose tissue vs white adipose tissue

Human AT is classified into two types based on morphological structure: WAT and BAT, both possessing the capacity to secrete unique adipokines. WAT consists of numerous adipocytes containing single lipid droplets and few mitochondria, storing excess energy as triglycerides for long-term storage. It exhibits sparse neural and vascular distribution (22). Adipocytes in BAT exhibit smaller intracellular lipid droplets, abundant mitochondria, and rich capillary and sympathetic nerve fiber structures. This structural foundation enables BAT to efficiently perform thermogenesis, with its core mechanism involving a special uncoupling protein-1 (UCP-1) abundant in mitochondria. which decouples oxidative phosphorylation of adenosine triphosphate (ATP), dissipating energy directly as heat (23, 24). Extensive past research has demonstrated that stimulating BAT thermogenesis significantly reduces obesity in mice and rats (25). BAT also enhances insulin sensitivity and regulates glucose homeostasis (26). Research indicates impaired BAT function and mass may contribute to metabolic and reproductive hormone dysregulation in women with PCOS, suggesting BAT activation holds therapeutic potential for improving PCOS symptoms by modulating gut microbiota (27). Excessive WAT is a well-established risk factor for metabolic diseases including diabetes, coronary heart disease, and metabolic syndrome (28–30). Recent therapeutic strategies have focused on promoting WAT “browning”—the process of beige adipocyte formation and UCP1 upregulation—to ameliorate metabolic disorders. This process correlates with FGF21-mediated improvements in weight loss and glucose homeostasis (31). Preclinical evidence confirms that adipokines regulate WAT browning (32), positioning beige AT as a promising therapeutic target for obesity, IR, and T2DM (32).Emerging clinical data support the translational potential of this approach: women with PCOS show reduced BAT activity that inversely correlates with IR severity (33, 34), while interventions like cold exposure, exercise, or β3-adrenergic agonists (e.g., mirabegron) have been shown to activate BAT and improve glucose metabolism in human studies (35–37). However, clinical translation for PCOS remains challenging due to off-target effects of systemic BAT activators (e.g., tachycardia), the relatively low abundance of functional BAT in adults, and a lack of large-scale PCOS-specific clinical trials. Despite these limitations, mechanistic studies continue to identify promising targets, such as angiotensin II-induced browning via AT2R-dependent activation of ERK1/2, Akt, and AMPK signaling pathways (38, 39). In summary, BAT activation and WAT browning represent a compelling therapeutic strategy for PCOS-associated metabolic dysfunction, though realizing its clinical potential requires further translational research addressing current limitations.

## 3 AT dysfunction

### 3.1 Definition of AT dysfunction and overview of potential mechanisms

Excessive accumulation of body fat is typically caused by nutrients exceeding the body’s requirements. These surplus nutrients are stored as triglycerides, commonly referred to as fat, while the cells storing triglycerides are called adipocytes, which constitute the primary components of AT. AT plays a pivotal role in maintaining metabolic homeostasis and systemic insulin sensitivity. It functions not only as a metabolic site but also as an endocrine organ, accounting for 2–70% of human body weight. Consequently, AT dysfunction triggers a cascade of metabolic disorders with systemic implications (40, 41). Broadly defined, AT dysfunction encompasses any alteration in AT anatomy, morphology (cell size or number), histology, or physiology that results in diminished insulin responsiveness, dysregulated adipokine secretion, and/or increased inflammation (30, 42). Current research suggests that the mechanisms underlying AT dysfunction primarily include: impaired AT storage capacity and hypoxia; AT hyperplasia and impaired lipogenesis; disrupted insulin signaling and glucose transport within AT; dysregulated lipolysis; and cytokine dysregulation with subacute inflammation within AT (43). AT dysfunction may also lead to altered adipokine levels (44). Adipokines are active hormones and other factors secreted by adipocytes, including adiponectin, apelin, chemerin, leptin, omentin, resistin, vaspin, and visfatin. Given their involvement in numerous critical physiological processes, adipokine dysregulation may contribute to endocrine disorders and has been identified as pivotal in the pathogenesis of obesity and obesity-related diseases. Studies suggest adipokine levels may serve as predictors for PCOS (45). Although multiple insults have been identified as contributing to AT dysfunction, AT inflammation and reduced insulin sensitivity appear to be common endpoints regardless of the initial injury.

### 3.2 Pathological drive of AT by androgens (positive feedback loop)

#### 3.2.1 IR axis dysfunctional

AT in obesity exhibits impaired proliferative remodeling, leading to adipocyte hypertrophy and systemic metabolic dysfunction (46), a phenomenon consistently observed in both animal and human studies (47). IR, a core feature of PCOS, is present in approximately 60% of normal-weight and 94% of obese affected women (48, 49). The correlation between enlarged subcutaneous adipocytes and reduced insulin sensitivity in PCOS patients, independent of BMI, indicates that adipocyte hypertrophy directly contributes to impaired insulin signaling (50). AT expansion induces local hypoxia, endoplasmic reticulum stress, and mitochondrial dysfunction (51, 52), triggering the release of reactive oxygen species and pro-inflammatory cytokines that promote systemic chronic inflammation, further exacerbating IR and enhancing lipolysis (53). Subsequently, elevated free fatty acids (FFAs) enter non-AT including liver, muscle, and pancreas, causing ectopic lipid deposition and associated lipotoxicity that additionally suppresses insulin signaling (54).Mechanistically, hyperinsulinemia promotes HA through three principal pathways: First, insulin directly stimulates the 17α-hydroxylase activity of P450c17 via activation of the PI3K signaling pathway (55), while indirectly enhancing ovarian androgen production by upregulating LH receptor expression (56). Second, hyperinsulinemia suppresses hepatic production of sex hormone-binding globulin (SHBG) and IGF-binding proteins, thereby increasing circulating free androgen and IGF-1 levels that further stimulate ovarian androgen synthesis (57, 58). Third, and notably, elevated expression and activity of AKR1C3 in AT of PCOS patients—this enzyme converts androstenedione to testosterone—creates a local insulin-androgen activation feedback loop that amplifies androgen production within AT itself (59, 60).

#### 3.2.2 Inflammation-endocrine axis

##### 3.2.2.1 Elevated inflammatory markers

PCOS is closely associated with low-grade chronic inflammation, with AT serving as a primary site of inflammatory activation. Compared to non-PCOS individuals, PCOS patients demonstrate elevated systemic inflammatory markers, including leukocytosis, increased C-reactive protein, and elevated pro-inflammatory cytokine levels (61). Obesity-associated AT inflammation is characterized by immune cell infiltration (e.g., macrophages) and secretion of inflammatory mediators (41, 62).The critical inflammation-androgen axis operates through defined molecular mechanisms: Adipose-derived inflammatory factors, particularly TNF-α and IL-6, activate the NF-κB signaling pathway in the ovary (63, 64). Activated NF-κB subsequently upregulates key steroidogenic proteins StAR and CYP17A1 (65). CYP17A1, as the only rate-limiting enzyme with dual catalytic activity in androgen synthesis, catalyzes the conversion of progesterone to androstenedione (66). In theca cells, androstenedione is further converted by AKR1C3 to more biologically active testosterone. Given the specific expression pattern of CYP17A1, theca cells represent the exclusive source of ovarian androgen production, and their overactivation is considered central to PCOS HA (67, 68). Furthermore, TNF-α has been demonstrated to directly stimulate theca cell proliferation and enhance androgen secretion (69), thereby reinforcing this inflammatory-endocrine axis in PCOS pathophysiology.

##### 3.2.2.2 Reduced adiponectin

PCOS patients exhibit marked adipokine dysregulation characterized by concurrent hypoadiponectinemia and a pro-inflammatory state (70). Adiponectin, an essential anti-inflammatory adipokine, plays a pivotal role in maintaining peripheral glucose and lipid homeostasis (71). At the molecular level, adiponectin primarily suppresses androgen synthesis through activation of two core signaling pathways: AMPK and PPAR-α. AMPK activation directly inhibits StAR protein expression, while PPAR-α activation primarily impedes mitochondrial cholesterol influx. These pathways act synergistically to restrict both the key substrate (cholesterol) and critical transport protein (StAR) required for androgen synthesis, thereby effectively reducing testosterone production (72, 73). Consequently, reduced adiponectin levels in PCOS patients attenuate this inhibitory effect on androgen synthesis, promoting the development and progression of HA. Notably, a bidirectional reinforcement exists between inflammation and HA: increases in pro-inflammatory factors coincide with decreases in anti-inflammatory factors. Importantly, inflammation induced by factors such as diet can directly trigger a hyperandrogenic state (74), further supporting the role of inflammation as a direct driver in PCOS pathogenesis.

##### 3.2.2.3 Leptin and neuroendocrine dysregulation

PCOS patients consistently demonstrate leptin dysregulation, characterized by hyperleptinemia that shows positive correlations with obesity severity, IR, and BMI (75). Leptin, a pro-inflammatory hormone produced primarily by white adipocytes, not only regulates energy balance through hypothalamic receptors (76, 77) but also plays a crucial role in immunometabolic crosstalk. The elevated leptin levels disrupt hypothalamic neuronal activity, particularly through inhibition of Kisspeptin neurons, leading to increased GnRH pulsatile secretion. This neuroendocrine alteration triggers excessive LH secretion and an elevated LH/FSH ratio. The heightened LH levels subsequently exert potent stimulation on ovarian theca cells, significantly enhancing their androgen synthesis capacity (78).

Notably, a bidirectional regulatory relationship exists between leptin and insulin: insulin serves as a key regulator of leptin production, and chronic hyperinsulinemia sustains elevated circulating leptin levels (79). This mutual reinforcement further amplifies the leptin-neuroendocrine axis’s stimulation of androgen synthesis, ultimately establishing a self-perpetuating vicious cycle in PCOS. Although the precise molecular mechanisms through which leptin influences GnRH pulsatility via Kisspeptin neurons remain controversial, its pivotal role in PCOS-related neuroendocrine dysfunction is well established.

## 4 HA

**Figure 2:**
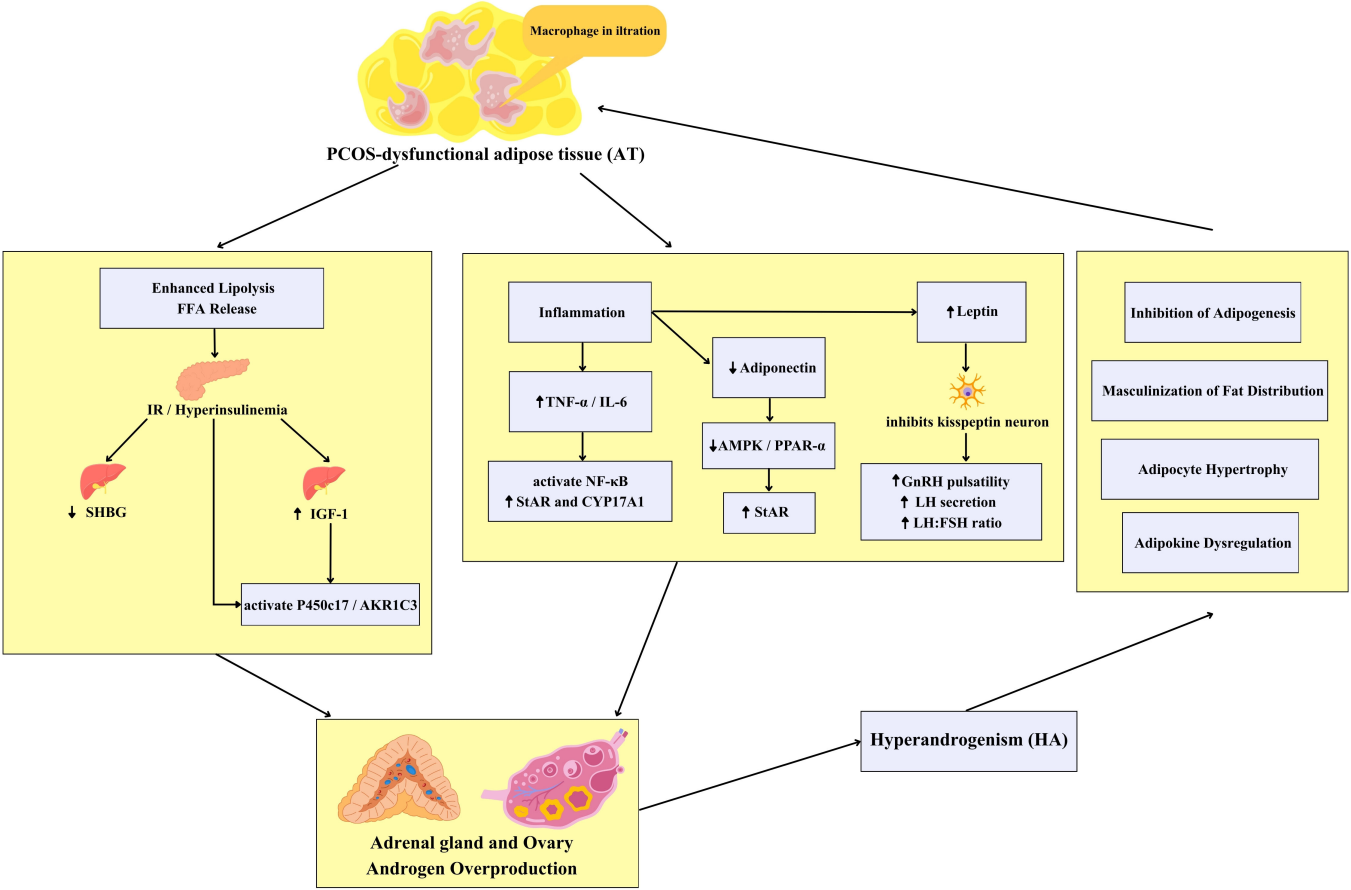
This model illustrates the self-reinforcing feedback loop between dysfunctional AT and HA that drives PCOS pathogenesis. The cycle initiates through three interconnected pathological pathways: enhanced lipolysis under IR releases free fatty acids that, together with elevated insulin/IGF-1 and reduced SHBG, stimulate ovarian theca cells to upregulate androgen-synthesizing enzymes P450c17 and AKR1C3; chronic inflammation characterized by elevated TNF-α/IL-6 activates NF-κB signaling to promote steroidogenic proteins StAR and CYP17A1 expression, while simultaneously inducing adipokine dysregulation through reduced adiponectin and elevated leptin. Critically, the decreased adiponectin levels attenuate its inhibitory effect on androgen synthesis, which is normally mediated through activation of the AMPK/PPAR-α signaling pathway and subsequent suppression of StAR expression - a key regulator of cholesterol transport into mitochondria for steroidogenesis. Concurrently, the increased leptin inhibits hypothalamic kisspeptin neurons, leading to amplified GnRH pulsatility, preferential LH secretion, and elevated LH/FSH ratio. The resulting HA then completes the loop by inducing masculinization of fat distribution, adipocyte hypertrophy, and further adipokine dysregulation that collectively exacerbate AT dysfunction. This continuous cross-talk between metabolic and endocrine tissues perpetuates the core features of PCOS. PCOS, Polycystic Ovary Syndrome; SHBG, Sex Hormone-Binding Globulin; IGF-1, Insulin-like Growth Factor-1; P450c17, Cytochrome P450 Family 17 Subfamily A; AKR1C3, Aldo-Keto Reductase Family 1 Member C3; TNF-α, Tumor Necrosis Factor-Alpha; IL-6, Interleukin-6; NF-κB, Nuclear Factor Kappa-Light-Chain-Enhancer of Activated B Cells; StAR, Steroidogenic Acute Regulatory Protein; CYP17A1, Cytochrome P450 Family 17 Subfamily A Member 1; AMPK, AMP-activated Protein Kinase; PPAR-α, Peroxisome Proliferator-Activated Receptor Alpha; GnRH, Gonadotropin-Releasing Hormone; LH, Luteinizing Hormone; FSH, Follicle-Stimulating Hormone.

Compared to women without PCOS, 60%–75% of PCOS patients exhibit elevated circulating androgen levels (80). HA is recognized as a hallmark feature of PCOS, and its central role in disease pathogenesis has been further supported by multiple *in vitro* experiments and animal model studies (81, 82).The primary source of HA in PCOS is the ovaries, with androstenedione and testosterone being the predominant types of androgens secreted. Decades of research indicate that the HA observed in PCOS women primarily stems from intrinsic functional abnormalities in ovarian theca cells (TC) (83). Although the ovaries are the primary site of excess androgen production, in approximately 20–30% of PCOS patients, the adrenal glands may also contribute to elevated androgen secretion (65). Elevated androgen levels in PCOS women promote the recruitment of primordial follicles, leading to impaired follicular maturation (65). Research by Bongrani et al. further indicates that PCOS-associated ovarian androgen excess manifests as a selective increase in TC androgen synthesis, accompanied by a relative decrease in adrenal androgen secretion (84). Another hypothesis suggests PCOS may originate from androgen exposure during critical embryonic periods or intrauterine growth restriction. Additionally, PCOS-associated HA is considered a potential underlying cause of AT inflammation (69) (**Figure 2**).

### 4.1 Regulation of AT by HA (reverse pathway)

HA plays a crucial role in the development of PCOS-related metabolic abnormalities. Evidence indicates that HA is closely associated with AT dysfunction (80). Androgens themselves regulate AT, influencing adipocyte distribution, differentiation, and metabolic function. Elevated androgen levels increase adipocyte size, disrupt lipogenesis and lipolysis, and impair the production of adipokines with insulin-sensitizing properties (e.g., adiponectin). These combined effects of androgen excess contribute to IR in PCOS, which further stimulates androgen secretion, creating a vicious cycle (84, 85). As previously discussed, AT dysfunction can lead to abnormal androgen secretion, thereby promoting the onset and progression of PCOS. Given that AT both responds to androgen signaling and possesses the capacity to synthesize and secrete androgens, a bidirectional regulatory relationship exists between AT and androgens. Under physiological conditions, AT contributes to hormonal homeostasis by storing and secreting androgens; however, excess androgens may act as an “amplifier,” further exacerbating AT dysfunction and promoting metabolic disorders.

#### 4.1.1 Impact on fat distribution

Androgens serve as key regulators of fat distribution, exerting a pronounced “masculinizing” remodeling effect on body fat patterning in PCOS patients. Imaging studies consistently demonstrate that HA drives a shift from the typical female gynoid (gluteofemoral) fat distribution toward a male-type central adiposity pattern, characterized by increased accumulation of abdominal and VAT alongside relative reduction in peripheral fat depots (86–88). This androgen-induced masculinization of fat distribution shows a positive correlation with circulating androgen levels and is similarly observed in female-to-male transgender individuals receiving testosterone therapy (89, 90), providing compelling human evidence for androgen-mediated fat redistribution. From a mechanistic perspective, androgens may promote VAT deposition through interactions with leptin signaling and energy expenditure pathways (91, 92), though the precise molecular mechanisms underlying this process require further elucidation. Critically, this androgen-driven alteration in fat distribution exacerbates PCOS-associated metabolic abnormalities, thereby contributing to a self-perpetuating vicious cycle of metabolic and endocrine dysfunction.

#### 4.1.2 Effects on adipocyte size and generation

Androgens exert significant effects on AT morphology and function, primarily by promoting adipocyte hypertrophy and inhibiting lipogenesis. Clinical studies reveal that adipocyte volume in PCOS patients is significantly larger than in BMI-matched controls, with adipocyte size positively correlated with circulating androgen levels (93). This finding is further validated in animal experiments: peripuberal female rats exposed to excessive androgens exhibit adipocyte hypertrophy in both subcutaneous and visceral fat depots, accompanied by developing IR (94). At the molecular level, androgens regulate AT biology through multiple pathways: enlarged adipocytes stimulate preadipocyte proliferation by releasing paracrine factors, while androgens directly inhibit the differentiation of preadipocytes into mature adipocytes (95–99). This phenomenon was confirmed in human primary adipocyte cultures, demonstrating that androgens simultaneously inhibit both the differentiation of adipose stem cells into preadipocytes and their subsequent maturation process (100, 101). On the other hand, androgens exert their anti-adipogenic effects by activating the androgen receptor. Specific mechanisms include: inhibiting the expression of key adipogenesis transcription factors such as PPARγ and C/EBPα induced by BMP4; and concurrently limiting PPAR-γ-mediated adipogenesis by competing for the coactivator ARA70 (102). These mechanisms collectively impair adipogenesis in PCOS patients, an effect partially reversible with anti-androgen therapy. Notably, hypertrophic adipocytes exhibit heightened inflammatory sensitivity and a propensity for macrophage infiltration, potentially further impairing insulin sensitivity and perpetuating a vicious cycle of metabolic dysfunction (70, 103).

#### 4.1.3 Effects on lipolysis and adipokine secretion

##### 4.1.3.1 Abnormal lipolysis

Another manifestation of AT dysfunction in PCOS women is dyslipolysis, which triggers ectopic lipid deposition and lipotoxicity (104). In PCOS patients, androgens suppress lipolysis through long-acting specific effects, particularly in SAT. This mechanism involves reduced expression of β2-adrenergic receptors, androgen-sensitive lipase (HSL)—the key enzyme mediating β-adrenergic lipolysis in WAT—and protein kinase A regulatory subunit IIβ (PKA-RegIIβ), thereby impairing catecholamine-stimulated lipolysis (105). Studies report that testosterone combined with a Western diet further suppresses β-adrenergic-stimulated lipolysis in WAT, accelerating AT dysfunction (106). Clinical experiments by Zang et al. also found that long-term testosterone intervention reduces HSL expression in SAT of postmenopausal women (107). Such androgen-mediated lipid metabolism abnormalities impair adipocyte lipid storage capacity and may promote IR. Another key factor in this lipolytic dysfunction is the elevated expression and activity of AKR1C3 in SCAT of PCOS patients (59). Studies indicate that AKR1C3 is the sole enzyme in AT capable of activating A4 into testosterone (108), and its enhanced activity leads to increased local testosterone production, thereby amplifying androgenic effects. O’Reilly et al. emphasized that AKR1C3 serves as a key regulator linking IR to androgen excess in PCOS. They found that selective *in vitro* inhibition of AKR1C3 significantly alleviated androgen-induced adipogenesis defects and PCOS-associated phenotypes (59).

##### 4.1.3.2 Adipokine dysregulation

Patients with PCOS exhibit significant dysregulation in adipokine secretion, characterized by reduced levels of insulin-sensitizing adiponectin and elevated levels of pro-inflammatory leptin. Extensive clinical evidence confirms that circulating adiponectin levels in PCOS patients are significantly lower than in healthy controls and negatively correlated with free testosterone levels (109, 110). Mechanistic studies indicate that androgens directly suppress adiponectin synthesis and secretion, a process considered a key pathway leading to IR in PCOS patients (111–113). Concurrently, PCOS patients commonly exhibit hyperleptinemia and leptin resistance. Research has shown that serum leptin levels correlate closely with the severity of HA and IR (114). From a pathophysiological perspective, a bidirectional regulatory relationship exists between leptin and androgens: on one hand, androgens promote elevated leptin levels; on the other hand, high leptin levels further exacerbate HA by inhibiting aromatase activity and reducing the conversion of androgens to estrogens (115, 116). Beyond modulating adiponectin and leptin, androgens also reduce levels of adipokines like retinol-like 1while elevating factors such as chemerin, follistatin, visfatin, and vaspin. These alterations may correlate with free testosterone levels in PCOS patients (111, 117). Extensive research indicates that pro-inflammatory and pro-fibrotic adipokines are universally elevated in PCOS patients, while adipokines that enhance insulin sensitivity, reduce inflammation, and support ovarian function are generally diminished. This imbalance collectively perpetuates and exacerbates HA and its associated metabolic and reproductive disorders.

## 5 Addressing AT dysfunction and hormone therapy for PCOS

### 5.1 Lifestyle interventions

Maintaining a healthy lifestyle throughout the PCOS lifecycle is crucial, focusing on overall wellness, preventing weight gain, and managing weight when necessary. Lifestyle modifications have been widely established as the primary treatment strategy for weight management in women with PCOS, particularly emphasizing the critical role of diet and physical activity in obesity management (118). Regarding exercise interventions, Mubarra et al. compared the effects of high-intensity interval training versus alternate-day strength training over 12 days, finding both reduced testosterone levels and body fat in PCOS patients, with HIIT demonstrating more pronounced effects (119). A systematic review encompassing 498 participants further demonstrated that lifestyle interventions can improve HA status and metabolic disorders in PCOS patients, thereby alleviating overall disease severity (120). Beyond exercise, multiple studies indicate that low-calorie diets (regardless of specific strategy) significantly improve insulin sensitivity, androgen levels, ovarian function, and fertility in obese women with PCOS (121–123). Moreover, in overweight or obese PCOS patients, combining a low-calorie diet with aerobic exercise more effectively optimizes body composition (e.g., greater fat mass reduction) and further alleviates HA-related symptoms (124).

### 5.2 Pharmacological treatment

#### 5.2.1 Oral contraceptive pills

Most PCOS patients fail to achieve significant clinical and biochemical improvements through lifestyle modifications alone, necessitating pharmacological intervention. OCPs represent the first-line drug therapy for PCOS. While no specific formulation is universally recommended, low-dose ethinyl estradiol preparations with fewer side effects are often prioritized in clinical practice (119). OCPs are typically taken continuously for 6 months. They effectively improve HA and alleviate PCOS-related symptoms by suppressing ovulation and preventing ovarian cyst formation (125). However, OCP use carries certain risks, including increased venous thrombosis and potential impacts on bone health (126, 127). Failure to strictly adhere to medical instructions during treatment may also cause abnormal uterine bleeding, necessitating standardized use under physician guidance.

#### 5.2.2 Metformin

As one of the most commonly used medications for PCOS, metformin is an insulin sensitizer. Research by Guan et al. indicates that metformin positively impacts reducing circulating androgens, increasing SHBG, and improving IR (128). Additional studies have found that metformin can indirectly influence AT function, such as elevating adiponectin levels and decreasing leptin (129). This medication also effectively improves ovulation rates (130). A multicenter randomized controlled trial involving 320 women with PCOS and anovulatory infertility demonstrated that adding metformin to standard infertility treatment increased pregnancy rates by 1.6-fold (131). Despite its widespread use in PCOS treatment, attention should be paid to its common gastrointestinal side effects.

#### 5.2.3 Thiazolidinediones

TZDs are also widely used in PCOS treatment. Their primary mechanism involves activating peroxisome proliferator-activated receptor gamma (PPAR-γ) (43). As a nuclear receptor, activation of PPAR-γ significantly improves insulin sensitivity in AT and skeletal muscle (132). Studies indicate that TZDs are associated with improvements in multiple reproductive parameters (133, 134). A 3-month randomized, double-blind, controlled trial randomly assigned 40 premenopausal women with PCOS to either pioglitazone (30 mg/day) or placebo. Results demonstrated that pioglitazone significantly improved insulin sensitivity, HA, and ovulation rates (135), further underscoring the significance of AT dysfunction in the pathophysiology of PCOS (136–138). It is important to note that TZDs can cross the placental barrier and are therefore contraindicated during pregnancy; caution is advised for PCOS women with reproductive aspirations.

#### 5.2.4 Glucagon-like peptide-1 receptor agonists

GLP-1 RAs, whether used monotherapy or in combination with metformin, improve glucose metabolism and IR. Furthermore, combination therapy with metformin and liraglutide demonstrates superior efficacy in improving reproductive abnormalities and HA compared to metformin monotherapy, primarily through regulating hypothalamic-pituitary-ovarian axis function (139). Liao et al. further demonstrated that for obese PCOS patients, GLP-1 RA combined with metformin outperformed oral contraceptive pills (OCPs) plus metformin in reducing BMI, improving metabolism, and promoting ovulation (140, 141). This evidence suggests GLP-1 RAs may represent a more suitable clinical treatment option for obese PCOS patients.

#### 5.2.5 Androgen antagonists

Androgen antagonists such as flutamide are primarily used to treat prostate cancer. Some studies indicate its efficacy in PCOS treatment, improving hirsutism, reducing androgen levels, and promoting ovulatory cycle restoration (111, 142). The findings from the study propose that in the pathophysiological cascade of PCOS, flutamide combined with metformin may prevent or reverse downstream effects by correcting upstream abnormalities such as hyperinsulinemia or HA (143). Other antiandrogens (e.g., cyproterone acetate, spironolactone, finasteride) may be used alone, but caution is warranted due to their potential to cause feminization in male fetuses; thus, they are only suitable for female patients requiring contraception. A study comparing monotherapy with metformin versus combination therapy with metformin plus spironolactone found that combination therapy may offer greater advantages in reducing BMI and serum androgen levels, though no significant synergistic effect was demonstrated overall (144).

### 5.3 Bariatric surgery

As an effective intervention for morbid obesity, bariatric surgery has also been found to significantly influence PCOS phenotypic characteristics and hormone levels. A retrospective analysis of 930 PCOS patients undergoing bariatric surgery demonstrated significant postoperative improvements in BMI, lipid profiles, and androgen levels. The study also noted reduced ovarian volume and suggested free testosterone as a potential predictor of metabolic benefits from surgery (145). The findings from the study further demonstrated that sleeve gastrectomy improves menstrual and hormonal status, promotes ovulation, and increases pregnancy rates (146). The precise mechanisms by which bariatric surgery improves metabolic and reproductive characteristics in PCOS patients remain incompletely understood, but may primarily result from post-operative weight loss-induced changes in AT function and improved IR, leading to reduced androgen levels and increased SHBG (147). Despite its significant efficacy, bariatric surgery is not typically the first-line treatment for PCOS due to its invasive nature and associated high surgical risks.

## 6 Emerging and potential therapeutic strategies

Given the pivotal role of AT dysfunction interacting with androgens in PCOS pathogenesis, disrupting this vicious cycle has emerged as a novel therapeutic direction, yielding multiple potential targets. SGLT-2 inhibitors, a novel class of hypoglycemic agents, demonstrate promising effects in significantly reducing body weight, improving HA, and alleviating IR. They are currently being explored for PCOS treatment, particularly for obese patients unresponsive to lifestyle interventions (148–150). Research on gut microbiota has advanced rapidly in recent years, with the “gut microbiota–AT–ovarian axis” emerging as a hotspot for improving systemic inflammation, IR, and HA, warranting further exploration (23). Additionally, BAT transplantation has demonstrated therapeutic potential in animal studies. Yuan et al. found that transplanting BAT into dehydroepiandrosterone-induced PCOS model rats significantly improved anovulation, HA, and polycystic ovarian status, restored regular estrous cycles, elevated systemic insulin sensitivity to normal levels, and enhanced fertility. Notably, BAT transplantation also activated endogenous BAT function, elevating circulating adiponectin levels, suggesting BAT transplantation may represent a promising therapeutic option for PCOS (151). Anti-TNF-α therapy has also been proposed as a potential treatment for PCOS. Animal studies demonstrate that TNF-α promotes testosterone synthesis in granulosa cells (e.g., KGN cells) by suppressing CYP19A1 expression, a process blocked by the TNF-α inhibitor etanercept (ETA) (152). These findings suggest ETA-mediated anti-TNF-α therapy may effectively alleviate PCOS by reducing excessive HA levels.

## 7 Discussion

In conclusion, this review delineates a compelling bidirectional interplay between dysfunctional AT and HA, which forms a self-perpetuating vicious cycle central to the pathogenesis of PCOS. This model provides a cohesive pathophysiological framework that explains the intricate link between metabolic and reproductive disturbances in the syndrome.

However, a truly critical synthesis must also acknowledge the heterogeneity of PCOS and the existence of contradicting evidence. Notably, studies focusing on lean PCOS populations demonstrate that HA can occur in the absence of overt AT dysfunction (153–155), suggesting that primary ovarian, adrenarche or neuroendocrine defects may initiate the cycle in certain phenotypes. This evidence does not invalidate the adipose-androgen axis but rather refines our understanding of its context, highlighting that it is likely the dominant driver in the metabolic phenotype, while other factors may predominate in leaner individuals (156–160). Therefore, modern PCOS management must transcend a fragmented, symptom-centric approach. Embracing this complex pathophysiology necessitates a paradigm shift towards integrated, multi-targeted systemic intervention strategies. Future therapeutic innovations will undoubtedly focus more precisely on directly targeting AT function and its secretory factors, paving the way for more precise and effective long-term management of this complex syndrome.
